# Bis[bis­(1,10-phenanthroline-κ^2^ *N*,*N*′)copper(I)] μ_6_-oxido-dodeca­kis-μ_2_-oxido-hexa­oxidohexa­tungsten(VI)

**DOI:** 10.1107/S1600536809020170

**Published:** 2009-06-06

**Authors:** Zhen-Fang Li, Bi-Song Zhang, Chang-Sheng Wu

**Affiliations:** a College of Materials Science and Chemical Engineering, Jinhua College of Profession and Technology, Jinhua, Zhejiang 321017, People’s Republic of China

## Abstract

The title compound, [Cu(C_12_H_8_N_2_)_2_]_2_[W_6_O_19_], consists of two [Cu(phen)_2_]^+^ cations (phen = 1,10-phenanthroline) and one typical [W_6_O_19_]^2−^ isopolyanion. The Cu^I^ atom is coordinated by four N atoms from two bidentate chelating phen ligands in a distorted tetra­hedral geometry. The hexa­tungstate anion, lying on an inversion center and possessing the well known Lindqvist structure, is formed by six edge-sharing WO_6_ octa­hedra, thus exhibiting an approximate *O_h_* symmetry. Three kinds of O atoms exist in the hexa­tungstate, *viz.* terminal O_*a*_, bridging O_*b*_ and central O_*c*_ atoms. Besides the electrostatic effects between the anions and cations, weak C—H⋯O hydrogen bonds exist between the phen ligands and O_*a*_ or O_*b*_ atoms. The mean inter­planar distances of 3.485 (1) and 3.344 (1) Å indicate π–π stacking inter­actions between neighboring phen ligands. These weak hydrogen bonds and π–π stacking inter­actions lead to a two-dimensional network.

## Related literature

For general background to hexa­tungstate compounds, see: Khan *et al.* (1998 ▶); Meng *et al.* (2006 ▶); Zhang *et al.* (2004 ▶). For related structures, see: Li & Zhang (2008 ▶); Zhang (2008 ▶).
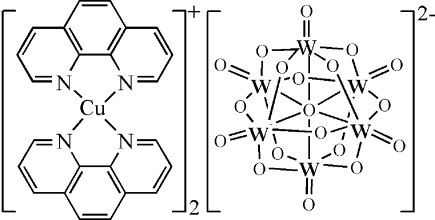


## Experimental

### 

#### Crystal data

[Cu(C_12_H_8_N_2_)_2_]_2_[W_6_O_19_]
*M* *_r_* = 2255.00
Triclinic, 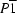

*a* = 10.364 (2) Å
*b* = 11.772 (2) Å
*c* = 11.899 (2) Å
α = 108.603 (3)°
β = 102.151 (3)°
γ = 100.694 (3)°
*V* = 1294.0 (4) Å^3^
*Z* = 1
Mo *K*α radiation
μ = 14.17 mm^−1^
*T* = 290 K
0.19 × 0.16 × 0.07 mm

#### Data collection

Bruker SMART APEX CCD diffractometer
Absorption correction: multi-scan (*SADABS*; Sheldrick, 1996 ▶) *T*_min_ = 0.09, *T*_max_ = 0.39
7111 measured reflections
4932 independent reflections
3737 reflections with *I* > 2σ(*I*)
*R*_int_ = 0.035

#### Refinement

*R*[*F* ^2^ > 2σ(*F* ^2^)] = 0.059
*wR*(*F* ^2^) = 0.157
*S* = 1.00
4932 reflections
376 parameters
H-atom parameters constrained
Δρ_max_ = 2.72 e Å^−3^
Δρ_min_ = −4.78 e Å^−3^

### 

Data collection: *SMART* (Bruker, 2007 ▶); cell refinement: *SAINT* (Bruker, 2007 ▶); data reduction: *SAINT*; program(s) used to solve structure: *SHELXS97* (Sheldrick, 2008 ▶); program(s) used to refine structure: *SHELXL97* (Sheldrick, 2008 ▶); molecular graphics: *SHELXTL* (Sheldrick, 2008 ▶); software used to prepare material for publication: *SHELXTL*.

## Supplementary Material

Crystal structure: contains datablocks I, global. DOI: 10.1107/S1600536809020170/hy2197sup1.cif

Structure factors: contains datablocks I. DOI: 10.1107/S1600536809020170/hy2197Isup2.hkl

Additional supplementary materials:  crystallographic information; 3D view; checkCIF report

## Figures and Tables

**Table 1:** Selected bond lengths (Å)

Cu1—N1	2.027 (14)
Cu1—N2	2.013 (11)
Cu1—N3	2.050 (12)
Cu1—N4	2.007 (11)
W1—O4	1.678 (10)
W1—O3^i^	1.904 (10)
W1—O1	1.926 (8)
W1—O9	1.929 (9)
W1—O8^i^	1.931 (8)
W1—O10	2.3139 (6)
W2—O2	1.672 (9)
W2—O3	1.904 (11)
W2—O6	1.915 (9)
W2—O1	1.923 (8)
W2—O5^i^	1.941 (9)
W2—O10	2.3314 (6)
W3—O7	1.691 (11)
W3—O5	1.899 (10)
W3—O9	1.907 (9)
W3—O6	1.912 (9)
W3—O8	1.921 (9)
W3—O10	2.3392 (6)

Symmetry code: (i) 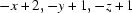.

**Table 2:** Hydrogen-bond geometry (Å, °)

*D*—H⋯*A*	*D*—H	H⋯*A*	*D*⋯*A*	*D*—H⋯*A*
C1—H1⋯O3^ii^	0.93	2.53	3.36 (2)	149
C17—H17⋯O4^iii^	0.93	2.52	3.45 (2)	178
C15—H15⋯O9^iii^	0.93	2.49	3.43 (1)	178

Symmetry codes: (ii) 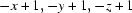; (iii) 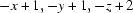.

**Fig. 1.**
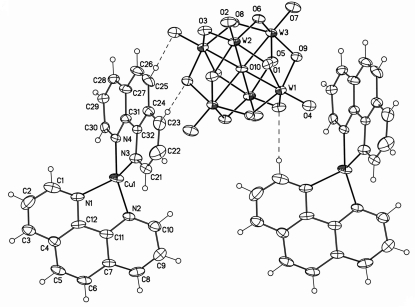
Molecular structure of the title compound. Displacement ellipsoids are drawn at the 50% probability level.

**Fig. 2.**
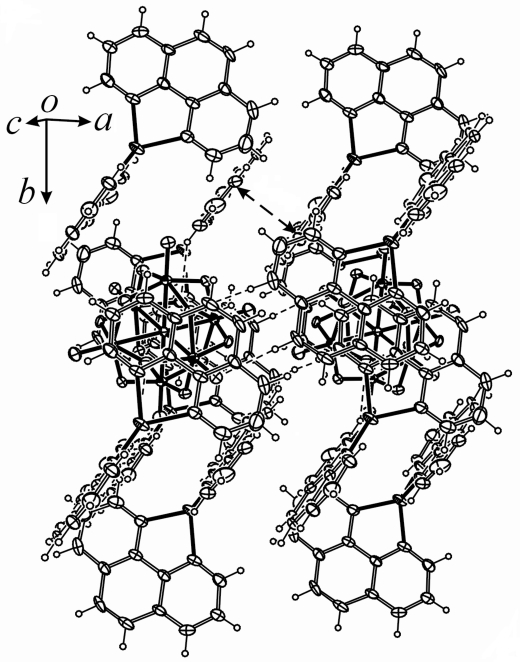
The π–π stacking interactions (dashed double arrows), with the mean interplanar distance of 3.485 (1) Å, and C—H···O hydrogen bonds (dashed lines) in the title compound.

**Fig. 3.**
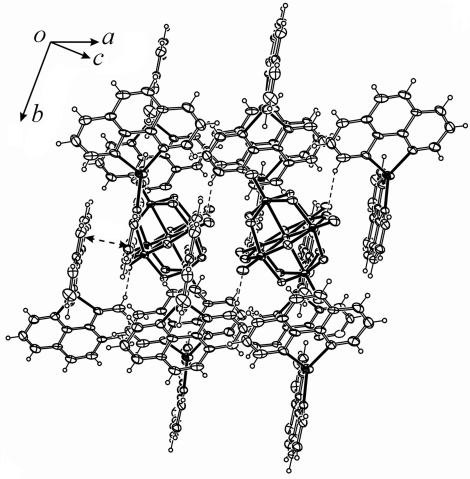
The π–π stacking interactions (dashed double arrows), with the mean interplanar distance of 3.344 (1) Å, and C—H···O hydrogen bonds (dashed lines) in the title compound.

## supplementary crystallographic information

### Comment

Organic–inorganic hybrid compounds comprise hexatungstate and organic components (Khan *et al.*, 1998; Meng *et al.*, 2006; Zhang *et al.*, 2004). In this context, we have studied and reported the crystal structures of dodecahydroxydodecatungsten henicosahydrate (Li & Zhang, 2008) and hexakis(3-hydroxo)tetra(2-hydroxo)octadeca(2-oxo)tetradecaoxodisodium(I) dodecatungsten dodecahydrate (Zhang, 2008). In this paper, we report the synthesis and structure of the title complex, [Cu(phen)_2_]_2_[W_6_O_19_].

The analysis of crystal structure shows that the title organic–inorganic hybrid compound consists of one hexatungstate cluster anion (W_6_O_19_)^2-^ and two monovalent coordination cations [Cu(phen)_2_]^+^ (Fig. 1). In the [Cu(phen)_2_]^+^ cation, the Cu^I^ atom is coordinated by four N atoms from two bidentate chelating phen ligands in a distorted tetrahedral geometry (Table 1). The dihedral angle of the two phen ligands is 104.9 (2)°, and the bond distances of Cu—N are in the range of 2.007 (11)—2.050 (12) Å. The hexatungstate (W_6_O_19_)^2-^ anion, lying on an inversion center and possessing the well-known lindqvist structure, is formed by six edge-sharing WO_6_ octahedra, thus exhibiting an approximate *O_h_* symmetry. Three kinds of O atoms exist in the hexatungstate, the ending O_a_ (O2, O4, O7), the bridging O_b_ (O1, O3, O5, O6, O8, O9) and the central O_c_ (O10) atoms. The bond lengths of W—O are obviously different, d(W—O_a_) = 1.672 (9)—1.691 (11)Å, d(W—O_b_) = 1.904 (10)—1.941 (9)Å, and d(W—O_c_) = 2.3139 (6)—2.3392 (6)Å. As we can see, the lengths of W—O_c_ are the longest and the W—O_a_ shortest. Besides the electrostatic effects between the anions and cations, the weak C—H···O hydrogen bonds exist between the phen ligands and O_a_ or O_b_ atoms (Fig.1, Fig.2, Fig.3 and Table 2). The mean interplanar distances of 3.485 (1) and 3.344 (1)Å indicate π–π stacking interactions between the neighboring phen ligands. These weak hydrogen bonds and π–π stacking interactions lead to a two-dimensional network.

### Experimental

A mixture of CuCO_3_ (0.124 g, 1.00 mmol), phen.H_2_O (0.050 g, 0.50 mmol), 2-chlorobenzoic acid (0.043 g, 0.25 mmol) and freshly prepared (NH_4_)_2_(WO_2_S_2_) (0.086 g, 0.27 mmol) in a ratio of 4:2:1:1 was added to CH_3_OH/H_2_O (1:2, v/v) mixed solution. After stirring for 2 h, the brown suspension obtained was sealed in a 50 ml Teflon-lined stainless steel vessel (degree of filling: 40%), heated to 393 K for 7 d and then naturally cooled to room temperature. The red crystals were collected, then washed with distilled water and dried in air.

### Refinement

H atoms were positioned geometrically and refined as riding atoms, with C—H = 0.93 Å and with *U*_iso_(H) = 1.2*U*_eq_(C). The largest peak in the final difference Fourier map is 0.96 Å from atom W3 and the deepest hole is 0.91 Å from atom W1.

### Crystal data

**Table:**

[Cu(C_12_H_8_N_2_)_2_]_2_[W_6_O_19_]	*Z* = 1
*M**_r_* = 2255.00	*F*(000) = 1030
Triclinic, *P*1	*D*_x_ = 2.894 Mg m^−^^3^
Hall symbol: -P 1	Mo *K*α radiation, λ = 0.71073 Å
*a* = 10.364 (2) Å	Cell parameters from 226 reflections
*b* = 11.772 (2) Å	θ = 1.9–26.0°
*c* = 11.899 (2) Å	µ = 14.17 mm^−^^1^
α = 108.603 (3)°	*T* = 290 K
β = 102.151 (3)°	Block, red
γ = 100.694 (3)°	0.19 × 0.16 × 0.07 mm
*V* = 1294.0 (4) Å^3^	

### Data collection

**Table:**

Bruker SMART APEX CCD diffractometer	4932 independent reflections
Radiation source: fine-focus sealed tube	3737 reflections with *I* > 2σ(*I*)
graphite	*R*_int_ = 0.035
φ and ω scans	θ_max_ = 26.0°, θ_min_ = 1.9°
Absorption correction: multi-scan (*SADABS*; Sheldrick, 1996)	*h* = −12→12
*T*_min_ = 0.09, *T*_max_ = 0.39	*k* = −14→14
7111 measured reflections	*l* = −7→14

### Refinement

**Table:**

Refinement on *F*^2^	Primary atom site location: structure-invariant direct methods
Least-squares matrix: full	Secondary atom site location: difference Fourier map
*R*[*F*^2^ > 2σ(*F*^2^)] = 0.059	Hydrogen site location: inferred from neighbouring sites
*wR*(*F*^2^) = 0.157	H-atom parameters constrained
*S* = 1.00	*w* = 1/[σ^2^(*F*_o_^2^) + (0.1032*P*)^2^] where *P* = (*F*_o_^2^ + 2*F*_c_^2^)/3
4932 reflections	(Δ/σ)_max_ = 0.001
376 parameters	Δρ_max_ = 2.72 e Å^−^^3^
0 restraints	Δρ_min_ = −4.78 e Å^−^^3^

### Fractional atomic coordinates and isotropic or equivalent isotropic displacement parameters (Å^2^)

**Table:**

	*x*	*y*	*z*	*U*_iso_*/*U*_eq_	
Cu1	0.3440 (2)	0.17655 (17)	0.7772 (2)	0.0595 (5)	
W1	0.99864 (5)	0.40295 (4)	0.64114 (5)	0.03513 (18)	
W2	0.79637 (5)	0.54681 (4)	0.52487 (5)	0.03706 (19)	
W3	1.12578 (5)	0.68912 (4)	0.66080 (5)	0.03581 (18)	
O1	0.8370 (8)	0.4620 (8)	0.6363 (8)	0.0341 (19)	
O2	0.6510 (10)	0.5825 (9)	0.5417 (12)	0.059 (3)	
O3	0.8394 (10)	0.6130 (8)	0.4065 (11)	0.050 (3)	
O4	0.9960 (11)	0.3340 (9)	0.7445 (10)	0.051 (3)	
O5	1.2619 (9)	0.6145 (8)	0.6099 (10)	0.045 (2)	
O6	0.9382 (9)	0.6879 (8)	0.6456 (9)	0.041 (2)	
O7	1.2177 (11)	0.8247 (9)	0.7782 (11)	0.061 (3)	
O8	1.1010 (8)	0.7305 (7)	0.5155 (8)	0.035 (2)	
O9	1.0969 (9)	0.5744 (8)	0.7415 (9)	0.039 (2)	
O10	1.0000	0.5000	0.5000	0.031 (3)	
N1	0.2642 (13)	0.0765 (10)	0.5929 (13)	0.048 (3)	
N2	0.4498 (12)	0.0484 (10)	0.7689 (11)	0.044 (3)	
N3	0.2306 (12)	0.2179 (10)	0.8990 (12)	0.047 (3)	
N4	0.3947 (12)	0.3613 (10)	0.8196 (10)	0.042 (3)	
C1	0.178 (2)	0.0906 (14)	0.504 (2)	0.068 (5)	
H1	0.1385	0.1560	0.5268	0.082*	
C2	0.142 (2)	0.0199 (17)	0.385 (2)	0.080 (6)	
H2	0.0773	0.0344	0.3278	0.096*	
C3	0.2024 (16)	−0.0766 (14)	0.3453 (16)	0.056 (4)	
H3	0.1787	−0.1268	0.2618	0.068*	
C4	0.2968 (15)	−0.0962 (12)	0.4310 (14)	0.044 (3)	
C5	0.3614 (17)	−0.1966 (13)	0.4029 (16)	0.054 (4)	
H5	0.3440	−0.2488	0.3208	0.065*	
C6	0.4458 (16)	−0.2159 (13)	0.4929 (15)	0.050 (4)	
H6	0.4824	−0.2835	0.4724	0.060*	
C7	0.4805 (13)	−0.1353 (11)	0.6187 (14)	0.039 (3)	
C8	0.5707 (16)	−0.1458 (14)	0.7184 (18)	0.058 (4)	
H8	0.6135	−0.2095	0.7022	0.069*	
C9	0.5981 (17)	−0.0672 (16)	0.8371 (18)	0.062 (4)	
H9	0.6565	−0.0772	0.9021	0.075*	
C10	0.5340 (17)	0.0312 (14)	0.8582 (16)	0.055 (4)	
H10	0.5523	0.0865	0.9391	0.066*	
C11	0.4206 (14)	−0.0325 (12)	0.6521 (14)	0.043 (3)	
C12	0.3243 (13)	−0.0200 (11)	0.5541 (15)	0.044 (4)	
C13	0.1527 (18)	0.1470 (15)	0.9380 (15)	0.057 (4)	
H13	0.1458	0.0622	0.9088	0.069*	
C14	0.082 (2)	0.189 (2)	1.017 (2)	0.081 (6)	
H14	0.0293	0.1342	1.0418	0.097*	
C15	0.0890 (16)	0.3146 (17)	1.0626 (15)	0.059 (4)	
H15	0.0407	0.3452	1.1178	0.071*	
C16	0.1710 (13)	0.3947 (13)	1.0228 (14)	0.044 (3)	
C17	0.1811 (16)	0.5272 (16)	1.0604 (14)	0.058 (4)	
H17	0.1343	0.5636	1.1145	0.069*	
C18	0.2586 (16)	0.5961 (14)	1.0160 (15)	0.058 (4)	
H18	0.2639	0.6805	1.0404	0.069*	
C19	0.3338 (14)	0.5465 (12)	0.9329 (15)	0.046 (4)	
C20	0.4137 (14)	0.6171 (12)	0.8855 (14)	0.048 (4)	
H20	0.4201	0.7015	0.9061	0.058*	
C21	0.4822 (15)	0.5605 (13)	0.8086 (14)	0.048 (3)	
H21	0.5372	0.6056	0.7762	0.057*	
C22	0.4684 (16)	0.4334 (14)	0.7791 (13)	0.047 (3)	
H22	0.5159	0.3967	0.7259	0.056*	
C23	0.3262 (14)	0.4188 (12)	0.8966 (14)	0.040 (3)	
C24	0.2409 (14)	0.3435 (12)	0.9414 (13)	0.040 (3)	

### Atomic displacement parameters (Å^2^)

**Table:**

	*U*^11^	*U*^22^	*U*^33^	*U*^12^	*U*^13^	*U*^23^
Cu1	0.0742 (13)	0.0385 (9)	0.0669 (13)	0.0301 (9)	0.0312 (11)	0.0058 (10)
W1	0.0357 (3)	0.0281 (3)	0.0464 (4)	0.0106 (2)	0.0163 (2)	0.0163 (3)
W2	0.0287 (3)	0.0320 (3)	0.0566 (4)	0.0150 (2)	0.0205 (3)	0.0157 (3)
W3	0.0350 (3)	0.0243 (3)	0.0448 (3)	0.0056 (2)	0.0146 (2)	0.0081 (2)
O1	0.035 (4)	0.039 (5)	0.043 (5)	0.012 (4)	0.024 (4)	0.025 (4)
O2	0.042 (6)	0.050 (6)	0.094 (9)	0.026 (5)	0.032 (6)	0.023 (6)
O3	0.045 (5)	0.032 (5)	0.076 (7)	0.013 (4)	0.019 (5)	0.020 (5)
O4	0.057 (6)	0.039 (5)	0.063 (7)	0.013 (4)	0.033 (5)	0.018 (5)
O5	0.032 (5)	0.033 (4)	0.064 (7)	0.004 (4)	0.009 (4)	0.015 (5)
O6	0.038 (5)	0.031 (4)	0.055 (6)	0.013 (4)	0.026 (4)	0.009 (4)
O7	0.061 (6)	0.037 (5)	0.077 (8)	0.003 (5)	0.030 (6)	0.009 (6)
O8	0.036 (5)	0.027 (4)	0.039 (5)	0.004 (3)	0.008 (4)	0.010 (4)
O9	0.042 (5)	0.030 (4)	0.045 (5)	0.007 (4)	0.018 (4)	0.013 (4)
O10	0.016 (5)	0.024 (5)	0.052 (8)	0.010 (4)	0.011 (5)	0.010 (6)
N1	0.050 (7)	0.034 (6)	0.067 (9)	0.025 (5)	0.022 (7)	0.014 (6)
N2	0.051 (7)	0.037 (6)	0.046 (7)	0.023 (5)	0.022 (6)	0.006 (6)
N3	0.048 (7)	0.037 (6)	0.058 (8)	0.017 (5)	0.019 (6)	0.013 (6)
N4	0.051 (7)	0.032 (5)	0.035 (6)	0.019 (5)	0.005 (5)	0.003 (5)
C1	0.073 (12)	0.034 (8)	0.090 (15)	0.007 (8)	0.036 (11)	0.008 (10)
C2	0.066 (11)	0.065 (12)	0.110 (18)	0.018 (9)	−0.004 (11)	0.051 (14)
C3	0.066 (10)	0.037 (8)	0.059 (10)	0.003 (7)	0.022 (9)	0.012 (8)
C4	0.053 (8)	0.027 (6)	0.050 (9)	0.000 (6)	0.025 (7)	0.014 (7)
C5	0.074 (11)	0.034 (7)	0.062 (10)	0.010 (7)	0.048 (9)	0.010 (8)
C6	0.063 (9)	0.037 (7)	0.068 (11)	0.028 (7)	0.046 (9)	0.017 (8)
C7	0.039 (7)	0.022 (6)	0.063 (9)	0.009 (5)	0.030 (7)	0.013 (6)
C8	0.052 (9)	0.048 (8)	0.090 (14)	0.031 (7)	0.035 (9)	0.027 (10)
C9	0.062 (10)	0.064 (10)	0.072 (12)	0.024 (8)	0.017 (9)	0.037 (10)
C10	0.071 (11)	0.045 (8)	0.053 (10)	0.023 (7)	0.026 (9)	0.011 (8)
C11	0.047 (8)	0.029 (6)	0.062 (9)	0.012 (5)	0.033 (7)	0.018 (7)
C12	0.039 (7)	0.025 (6)	0.080 (11)	0.015 (5)	0.036 (7)	0.019 (7)
C13	0.075 (11)	0.043 (8)	0.047 (9)	0.018 (8)	0.012 (8)	0.010 (8)
C14	0.085 (14)	0.088 (14)	0.109 (17)	0.035 (11)	0.059 (13)	0.061 (14)
C15	0.055 (9)	0.088 (12)	0.050 (10)	0.035 (9)	0.032 (8)	0.025 (10)
C16	0.035 (7)	0.046 (8)	0.046 (8)	0.021 (6)	0.011 (6)	0.007 (7)
C17	0.055 (9)	0.068 (10)	0.043 (9)	0.031 (8)	0.024 (8)	−0.005 (8)
C18	0.058 (9)	0.041 (8)	0.059 (10)	0.027 (7)	0.012 (8)	−0.004 (8)
C19	0.042 (7)	0.031 (7)	0.057 (10)	0.018 (6)	0.012 (7)	0.002 (7)
C20	0.052 (8)	0.029 (7)	0.053 (9)	0.008 (6)	0.001 (7)	0.012 (7)
C21	0.058 (9)	0.040 (7)	0.047 (9)	0.022 (7)	0.016 (7)	0.013 (7)
C22	0.064 (9)	0.053 (9)	0.031 (8)	0.031 (7)	0.016 (7)	0.016 (7)
C23	0.041 (7)	0.038 (7)	0.046 (8)	0.023 (6)	0.014 (6)	0.013 (7)
C24	0.050 (8)	0.034 (6)	0.035 (7)	0.022 (6)	0.008 (6)	0.008 (6)

### Geometric parameters (Å, °)

**Table:**

Cu1—N1	2.027 (14)	C3—H3	0.9300
Cu1—N2	2.013 (11)	C4—C12	1.39 (2)
Cu1—N3	2.050 (12)	C4—C5	1.45 (2)
Cu1—N4	2.007 (11)	C5—C6	1.34 (2)
W1—O4	1.678 (10)	C5—H5	0.9300
W1—O3^i^	1.904 (10)	C6—C7	1.42 (2)
W1—O1	1.926 (8)	C6—H6	0.9300
W1—O9	1.929 (9)	C7—C8	1.40 (2)
W1—O8^i^	1.931 (8)	C7—C11	1.444 (18)
W1—O10	2.3139 (6)	C8—C9	1.35 (2)
W2—O2	1.672 (9)	C8—H8	0.9300
W2—O3	1.904 (11)	C9—C10	1.42 (2)
W2—O6	1.915 (9)	C9—H9	0.9300
W2—O1	1.923 (8)	C10—H10	0.9300
W2—O5^i^	1.941 (9)	C11—C12	1.43 (2)
W2—O10	2.3314 (6)	C13—C14	1.34 (2)
W3—O7	1.691 (11)	C13—H13	0.9300
W3—O5	1.899 (10)	C14—C15	1.38 (3)
W3—O9	1.907 (9)	C14—H14	0.9300
W3—O6	1.912 (9)	C15—C16	1.41 (2)
W3—O8	1.921 (9)	C15—H15	0.9300
W3—O10	2.3392 (6)	C16—C24	1.383 (19)
N1—C1	1.31 (2)	C16—C17	1.46 (2)
N1—C12	1.393 (15)	C17—C18	1.34 (2)
N2—C10	1.321 (19)	C17—H17	0.9300
N2—C11	1.342 (18)	C18—C19	1.43 (2)
N3—C13	1.310 (19)	C18—H18	0.9300
N3—C24	1.377 (17)	C19—C20	1.39 (2)
N4—C22	1.308 (18)	C19—C23	1.407 (18)
N4—C23	1.363 (17)	C20—C21	1.36 (2)
C1—C2	1.33 (3)	C20—H20	0.9300
C1—H1	0.9300	C21—C22	1.39 (2)
C2—C3	1.40 (3)	C21—H21	0.9300
C2—H2	0.9300	C22—H22	0.9300
C3—C4	1.37 (2)	C23—C24	1.436 (19)
			
N4—Cu1—N2	134.8 (5)	C13—N3—C24	118.4 (13)
N4—Cu1—N1	113.4 (5)	C13—N3—Cu1	131.6 (10)
N2—Cu1—N1	83.1 (5)	C24—N3—Cu1	110.1 (10)
N4—Cu1—N3	83.3 (5)	C22—N4—C23	115.1 (11)
N2—Cu1—N3	122.9 (5)	C22—N4—Cu1	132.8 (10)
N1—Cu1—N3	124.7 (5)	C23—N4—Cu1	111.7 (9)
O4—W1—O3^i^	105.4 (5)	N1—C1—C2	125.8 (17)
O4—W1—O1	102.4 (4)	N1—C1—H1	117.1
O3^i^—W1—O1	152.1 (4)	C2—C1—H1	117.1
O4—W1—O9	103.8 (5)	C1—C2—C3	119.4 (18)
O3^i^—W1—O9	87.0 (4)	C1—C2—H2	120.3
O1—W1—O9	84.9 (4)	C3—C2—H2	120.3
O4—W1—O8^i^	103.6 (4)	C4—C3—C2	119.1 (16)
O3^i^—W1—O8^i^	86.7 (4)	C4—C3—H3	120.5
O1—W1—O8^i^	88.4 (4)	C2—C3—H3	120.5
O9—W1—O8^i^	152.6 (4)	C3—C4—C12	117.3 (14)
O4—W1—O10	179.0 (4)	C3—C4—C5	124.4 (14)
O3^i^—W1—O10	75.4 (3)	C12—C4—C5	118.2 (14)
O1—W1—O10	76.7 (2)	C6—C5—C4	121.2 (14)
O9—W1—O10	75.8 (3)	C6—C5—H5	119.4
O8^i^—W1—O10	76.8 (2)	C4—C5—H5	119.4
O2—W2—O3	104.0 (5)	C5—C6—C7	121.4 (13)
O2—W2—O6	104.2 (5)	C5—C6—H6	119.3
O3—W2—O6	85.8 (4)	C7—C6—H6	119.3
O2—W2—O1	104.6 (5)	C8—C7—C6	125.5 (13)
O3—W2—O1	151.4 (4)	C8—C7—C11	114.8 (13)
O6—W2—O1	86.5 (4)	C6—C7—C11	119.8 (14)
O2—W2—O5^i^	105.0 (5)	C9—C8—C7	122.9 (14)
O3—W2—O5^i^	85.8 (4)	C9—C8—H8	118.6
O6—W2—O5^i^	150.8 (4)	C7—C8—H8	118.6
O1—W2—O5^i^	87.6 (4)	C8—C9—C10	117.1 (16)
O2—W2—O10	178.9 (4)	C8—C9—H9	121.4
O3—W2—O10	75.0 (3)	C10—C9—H9	121.4
O6—W2—O10	75.4 (3)	N2—C10—C9	123.5 (15)
O1—W2—O10	76.4 (2)	N2—C10—H10	118.3
O5^i^—W2—O10	75.4 (3)	C9—C10—H10	118.3
O7—W3—O5	103.7 (5)	N2—C11—C12	119.9 (12)
O7—W3—O9	103.7 (5)	N2—C11—C7	123.1 (14)
O5—W3—O9	87.3 (4)	C12—C11—C7	117.0 (13)
O7—W3—O6	105.1 (5)	C4—C12—N1	123.4 (15)
O5—W3—O6	151.2 (4)	C4—C12—C11	122.2 (12)
O9—W3—O6	86.1 (4)	N1—C12—C11	114.4 (13)
O7—W3—O8	104.4 (5)	N3—C13—C14	124.0 (16)
O5—W3—O8	87.2 (4)	N3—C13—H13	118.0
O9—W3—O8	151.9 (4)	C14—C13—H13	118.0
O6—W3—O8	85.6 (4)	C13—C14—C15	120.0 (17)
O7—W3—O10	179.2 (4)	C13—C14—H14	120.0
O5—W3—O10	75.9 (3)	C15—C14—H14	120.0
O9—W3—O10	75.6 (3)	C14—C15—C16	118.1 (15)
O6—W3—O10	75.3 (3)	C14—C15—H15	120.9
O8—W3—O10	76.3 (2)	C16—C15—H15	120.9
W2—O1—W1	117.0 (4)	C24—C16—C15	118.1 (13)
W2—O3—W1^i^	119.4 (5)	C24—C16—C17	119.0 (14)
W3—O5—W2^i^	118.7 (4)	C15—C16—C17	122.9 (14)
W3—O6—W2	119.3 (4)	C18—C17—C16	119.1 (13)
W3—O8—W1^i^	117.0 (4)	C18—C17—H17	120.5
W3—O9—W1	118.4 (5)	C16—C17—H17	120.5
W1—O10—W1^i^	180.000 (1)	C17—C18—C19	123.5 (13)
W1—O10—W2	89.885 (19)	C17—C18—H18	118.3
W1^i^—O10—W2	90.115 (19)	C19—C18—H18	118.3
W1—O10—W2^i^	90.115 (19)	C20—C19—C23	118.0 (14)
W1^i^—O10—W2^i^	89.885 (19)	C20—C19—C18	123.8 (13)
W2—O10—W2^i^	180.00 (3)	C23—C19—C18	118.2 (14)
W1—O10—W3	90.18 (2)	C21—C20—C19	118.8 (12)
W1^i^—O10—W3	89.82 (2)	C21—C20—H20	120.6
W2—O10—W3	89.97 (2)	C19—C20—H20	120.6
W2^i^—O10—W3	90.03 (2)	C20—C21—C22	118.7 (14)
W1—O10—W3^i^	89.82 (2)	C20—C21—H21	120.7
W1^i^—O10—W3^i^	90.18 (2)	C22—C21—H21	120.7
W2—O10—W3^i^	90.03 (2)	N4—C22—C21	125.8 (14)
W2^i^—O10—W3^i^	89.97 (2)	N4—C22—H22	117.1
W3—O10—W3^i^	180.00 (2)	C21—C22—H22	117.1
C1—N1—C12	115.1 (14)	N4—C23—C19	123.7 (13)
C1—N1—Cu1	133.2 (10)	N4—C23—C24	117.5 (11)
C12—N1—Cu1	111.5 (10)	C19—C23—C24	118.8 (13)
C10—N2—C11	118.6 (12)	N3—C24—C16	121.4 (13)
C10—N2—Cu1	130.3 (10)	N3—C24—C23	117.2 (12)
C11—N2—Cu1	111.0 (10)	C16—C24—C23	121.4 (12)
			
O6—W2—O1—W1	−77.3 (5)	O3—W2—O6—W3	−76.6 (6)
O5^i^—W2—O1—W1	74.1 (5)	O1—W2—O6—W3	75.8 (5)
O3^i^—W1—O1—W2	4.5 (11)	O5^i^—W2—O6—W3	−3.0 (12)
O9—W1—O1—W2	78.0 (5)	O10—W2—O6—W3	−1.0 (4)
O8^i^—W1—O1—W2	−75.4 (5)	O7—W3—O8—W1^i^	179.1 (5)
O6—W2—O3—W1^i^	75.4 (6)	O5—W3—O8—W1^i^	75.6 (5)
O5^i^—W2—O3—W1^i^	−76.6 (6)	O9—W3—O8—W1^i^	−3.3 (11)
O9—W3—O5—W2^i^	76.2 (6)	O6—W3—O8—W1^i^	−76.5 (5)
O6—W3—O5—W2^i^	−0.6 (12)	O7—W3—O9—W1	−178.3 (5)
O8—W3—O5—W2^i^	−76.3 (6)	O5—W3—O9—W1	−74.9 (5)
O7—W3—O6—W2	−178.3 (6)	O6—W3—O9—W1	77.1 (5)
O9—W3—O6—W2	−75.1 (6)	O4—W1—O9—W3	179.6 (5)
O8—W3—O6—W2	78.1 (5)	O3^i^—W1—O9—W3	74.5 (6)
O2—W2—O6—W3	180.0 (6)	O1—W1—O9—W3	−78.9 (5)

Symmetry codes: (i) −*x*+2, −*y*+1, −*z*+1.

### Hydrogen-bond geometry (Å, °)

**Table:**

*D*—H···*A*	*D*—H	H···*A*	*D*···*A*	*D*—H···*A*
C1—H1···O3^ii^	0.93	2.53	3.36 (2)	149
C17—H17···O4^iii^	0.93	2.52	3.45 (2)	178
C15—H15···O9^iii^	0.93	2.49	3.43 (1)	178

Symmetry codes: (ii) −*x*+1, −*y*+1, −*z*+1; (iii) −*x*+1, −*y*+1, −*z*+2.
